# The Consumption of Energy Drinks Induces Blood-Brain Barrier Dysfunction in Wild-Type Mice

**DOI:** 10.3389/fnut.2021.668514

**Published:** 2021-05-03

**Authors:** Liam Graneri, Virginie Lam, Zachary D'Alonzo, Michael Nesbit, John C. L. Mamo, Ryusuke Takechi

**Affiliations:** ^1^Curtin Health Innovation Research Institute, Curtin University, Bentley, WA, Australia; ^2^Curtin Medical School, Faculty of Health Science, Curtin University, Bentley, WA, Australia; ^3^School of Population Health, Faculty of Health Science, Curtin University, Bentley, WA, Australia

**Keywords:** energy drinks, metabolic syndrome, blood-brain barrier, neurovascular integrity, epithelial function

## Abstract

Energy drinks containing significant quantities of caffeine and sugar are increasingly consumed, particularly by adolescents and young adults. Chronic ingestion of energy drinks may potentially regulate vascular risk factors. This study investigated the effects of chronic ingestion of energy drinks on blood-brain barrier (BBB) integrity and neuroinflammation. Male C57BL/6J mice were maintained on water (control), Mother^TM^ (ED), sugar-free Mother^TM^ (sfED), or Coca Cola^TM^ soft drink (SD) for 13 weeks. The BBB integrity and neuroinflammation were analyzed with semi-quantitative immunofluorescent microscopy. Blood pressure, plasma inflammatory cytokine levels and blood glucose were also considered. Following 13 weeks of intervention, mice treated with ED, sfED, and SD showed significant disruption of BBB. However, marked neuroinflammation was observed only in sfED group mice. The consumption of ED and sfED significantly altered the blood pressure and plasma concentrations of inflammatory cytokines, TNF-a, IL-4, IL-6, and IL-10, and both increased plasma glucose. Correlation analyses showed significant associations between BBB dysfunction and hypotension, hyperglycaemia and cytokine dyshomeostasis. The intake of energy drink, particularly the sugar free formulation, may compromise the integrity of BBB and induce neuroinflammation via hypotension, hyperglycaemia and inflammatory pathways.

## Introduction

The blood-brain barrier (BBB) describes the anatomical structure which surrounds the endothelial cells of cerebral capillaries (1). Its function is to serve as a selectively permeable barrier for brain parenchyme (2) and this is achieved ordinarily through highly regulated expression of endothelial tight junctions within the paracellular spaces. A disruption in BBB integrity permits the cerebral extravasation of plasma-borne molecules, which may activate astrocytes and microglia and promote neuroinflammation (3). Chronically exaggerated astrocytosis and microgliosis lead to heightened oxidative stress, which consequently promotes degeneration of neurons (4). Indeed, BBB dysfunction is commonly observed in various neurodegenerative disorders such as Alzheimer's disease and multiple sclerosis, suggesting a causal association (5–8).

Energy drinks are highly caffeinated, carbonated beverages first marketed in Europe and Asia in the 1960's (9). The primary ingredients with popular energy drinks such as Red Bull^TM^, Mother^TM^, and Monster^TM^ include caffeine/guarana [~160 mg per serving (375 ml)], remarkable abundance of taurine (~2,000 mg per serving), B6/B12 vitamins (1.0 mg/0.5 μg per serving, respectively), and in sugar-formulated energy drinks (~51 g sucrose per serving). Recent population studies report significantly accelerating ingestion trends of energy drinks, particularly in young adults (10). Due to their complex formulations, both negative and positive effects on BBB integrity may be realized with regular, significant consumption of energy drinks. Evidence consistent with the latter are findings that energy drinks can have acute hypertensive effects on blood pressure, but paradoxically, hypotensive effects with chronic ingestion (11–17). Chronic consumption of sugar-formulated energy drinks may also have secondary indirect neurovascular effects, exacerbating hyperglycaemia, insulin resistance (18–22) and neurovascular integrity and neuroinflammation (23–25).

To gain better insight into the potential effects of energy drinks on BBB integrity, in this pilot study, genetically unmanipulated wild-type mice were chronically provided with diluted energy drinks as their sole drinking solution. The putative effects of Mother^TM^, Sugar-free Mother^TM^, and Coca-Cola^TM^ (which lacks taurine, B-group vitamins and has substantially less caffeine) were studied in the context of neurovascular integrity and neuroinflammation.

## Methods

### Animals and Dietary Intervention

Wild-type C57BL/6J male mice were purchased at 5 weeks of age from the Animal Resources Center (WA, Australia). After 1 week of acclimatization period, mice were randomly assigned to one of 4 drink intervention groups (*n* = 10 per group) for 13 weeks, each given a unique drink intervention. The control group was given only water. The drink groups were given either Mother^TM^ energy drink (ED), sugar-free Mother^TM^ (sfED), or Coca-Cola^TM^ carbonated soft drink (SD); all diluted to 30% (v/v) in water. The age of mice and the study duration were determined based on previous studies where dietary intervention and cerebrovascular integrity were investigated (7, 26). The age of mice tested here is roughly equivalent to adolescent to young adult in human. The dose of each drink ingested was determined based on similar previous studies (27) in attempt to replicate the higher level of social relevance in the context of energy drink consumption. Male mice were chosen because energy drink consumption is more popular in men than women (28), and to be consistent with previous studies (26, 29). All groups were provided with standard rodent maintenance chow (AIN-93M, Specialty Feeds, WA, Australia). The major nutritional components of AIN-93M chow, Mother^TM^, sugar-free Mother^TM^, and Coca-Cola^TM^ are presented in Supplementary Table 1. The consumption of respective food and drink quantities were measured twice weekly. All animals were kept in individually ventilated cages with 12 h light/dark cycles, under controlled temperature (21°C) and air pressure. All drinks and diets were available *ad libitum*. All animal protocols described in this study were approved by Curtin University Animal Ethics Committee (approval no. ARE2018-3).

### Blood Pressure Measurement

At baseline and after 13 weeks of the drink intervention, systolic and diastolic blood pressures as well as mean arterial pressure were measured by using a CODA high-throughput tail cuff blood pressure system as described previously (30). Briefly, the mice were restrained, equipped with the tail cuff connected to CODA blood pressure reader and rested on an infrared heating pad. After 5 acclimatization trials, 15 readings of each blood pressure measure were taken with 10 s intervals between the measurements. The results are presented as a mean of all valid readings of the 15 measurements. The mean change of blood pressure from baseline to the 13 weeks per individual mouse is presented.

### Brain and Blood Sample Collection

Following the blood pressure analysis, the mice were anesthetized with gaseous isoflurane and blood samples were collected via cardiac puncture. Whole blood was collected, allowed to clot at room temperature for 30 min and centrifuged for 10 min at 4,000 rpm. Thereafter, the supernatant serum was aliquoted and stored at −80°C for further analysis. Each mouse brain was carefully removed, and the right hemisphere was fixed in 4% paraformaldehyde for 24 h and subsequently cryoprotected with 20% sucrose for 72 h before freezing in isopentane/dry ice and stored at −80°C for immuno-microscopy analyses.

### Assessment of BBB Integrity

The integrity of cerebral capillaries was assessed using an established protocol of blood-borne immunoglobulin-G (IgG) extravasation into the brain peri-vascular parenchymal space as an indication of non-specific “leakage” across the BBB (7, 26, 31). Briefly, 20 μm thick cryosections were blocked with 10% goat serum in PBS for 30 min and subsequently incubated with goat anti-mouse IgG conjugated with Alexa488 fluorophore (1:200, ThermoFisher, USA) for 20 h in the dark at 4°C. Thereafter, the nuclei were counterstained with DAPI (1:500) and mounted with antifade mounting media. The slides were viewed under a confocal microscope (UltraVIEW Vox, PerkinElmer). On average, 11 3-D images were captured, respectively, in the hippocampus and cortex of each brain section at 20× magnification to cover the majority of each brain region; and each 3-D image consisted of 20 Z-slices with 1 μm z-axis distance. The image capturing conditions (i.e., laser power, camera sensitivity and exposure time) were kept consistent amongst all animals of all experimental groups. The abundance of IgG extravasation was determined semi-quantitatively per image by measuring the voxel intensity of Alexa dye in the areas of peri-vascular parenchymal leakage using Volocity (PerkinElmer) as detailed previously (7). Briefly, the area of IgG extravasation (diffuse weak staining around the vessels) was selected with a magic wand tool of the software, which determines the voxel intensity of the selected region of interest. The experienced investigators identified the vessel area based on the intense IgG staining and endothelial nuclei staining and excluded these areas from the quantitation. The investigators were blinded to the treatment groups to eliminate any bias to these analyses.

### Analyses of Neuroinflammation

Neuroinflammation was assessed by determining ionized calcium-binding adapter molecule 1 (Iba-1) expression through quantitative immunofluorescent microscopy as established previously (32). Twenty μm sections underwent a 20 h antigen retrieval at 37°C in Tris-EDTA buffer. The sections were then rehydrated in PBS before being blocked in 10% goat serum in PBS for 30 min, and subsequently incubated with rabbit anti-mouse Iba-1 (1:500, WAKO, USA) for 20 h at 4°C. The sections were then incubated with goat anti-rabbit IgG conjugated with Alexa 488 fluorophore (1:500, ThermoFisher) for 2 h in the dark at 20°C. The slides were viewed and imaged with UltraVIEW Vox in the same manner as for the IgG staining protocol above. Fluorescence was quantitated with Volocity (ver 5.4.2) based on pre-set threshold parameters to ensure adequate overall fit for intensity threshold of all sections.

### Determination of Inflammatory Cytokines in Serum

Serum concentrations of inflammatory cytokines interleukin (IL)-2, IL-4, IL-6, IL-10, and tumor necrosis factor (TNF)-α were determined using a cytometric bead assay by using a commercial kit (BD Biosciences MA, USA) as reported previously (33). Briefly, 10 μl of undiluted serum, 10 μl of capture beads mixture, and 10 μl of PE detection reagent were mixed in a 96-well microplate and incubated in the dark for 2 h. After a wash, and centrifugation, the beads were resuspended (7) in 200 μl wash buffer. Results were acquired using FACS Canto II (BD Biosciences) and the data was analyzed by using FlowJo (FlowJo, OR, USA). The standards provided in the kit were used to construct a one-phase decay non-linear regression line that was subsequently used to interpolate the sample results.

### Statistical Methods

Based on previous similar studies, power calculation was done with effect size *d* = 1.1, α at 0.3, and power = 0.9, which indicated 10 mice per group to provide sufficient power (26). The data for each measure were inputted into GraphPad Prism (ver. 7.04 Graphpad Software, CA, US) and expressed as mean±SEM. Data normality was tested with D'Agostino-Pearson omnibus normality test. Statistical analysis was conducted using a one-way ANOVA for with Fisher's LSD *post-hoc* multiple comparison test for normally distributed data, whilst non-parametric Kruskal-Wallis test with Dunn's *post-hoc* test was used for the data without normal distribution. Statistical significance was determined at *p* < 0.05. Associations between BBB dysfunction or neuroinflammation and blood pressure or plasma inflammatory cytokines were assessed with Pearson's correlation analysis for the data that are normally distributed. For the data that were not normally distributed, non-parametric Spearman's correlation coefficient analysis was used.

## Results

The putative effects of chronic ingestion of either ED or sfED on neurovascular integrity and neuroinflammation were determined in wild-type C57BL/6J male mice. Mice were provided *ad-libitum* with diluted ED or sfED that were physiologically relevant. Consumption of chow, drink and energy intake are provided in Supplementary Figures 1A,B. The drink interventions were well-tolerated, and no adverse events were indicated. A substantially larger ingestion of drinking solutions were indicated in ED, sfED and SD groups compared to mice provided with water. However, the cumulative energy intake over the intervention period was comparable between treatments, due to a compensatory decrease in chow consumption in ED and SD treated mice (Supplementary Figure 1C). At 13 weeks of intervention, the body weight of the mice maintained on ED, sfED and SD were comparable to the control (Supplementary Figure 1D). Mice maintained on ED and SD were hyperlgycemic, however surprisingly, a similar increase in blood glucose was also realized in the mice that received sfED for 13 weeks (Supplementary Figure 1E).

**Figure 1:**
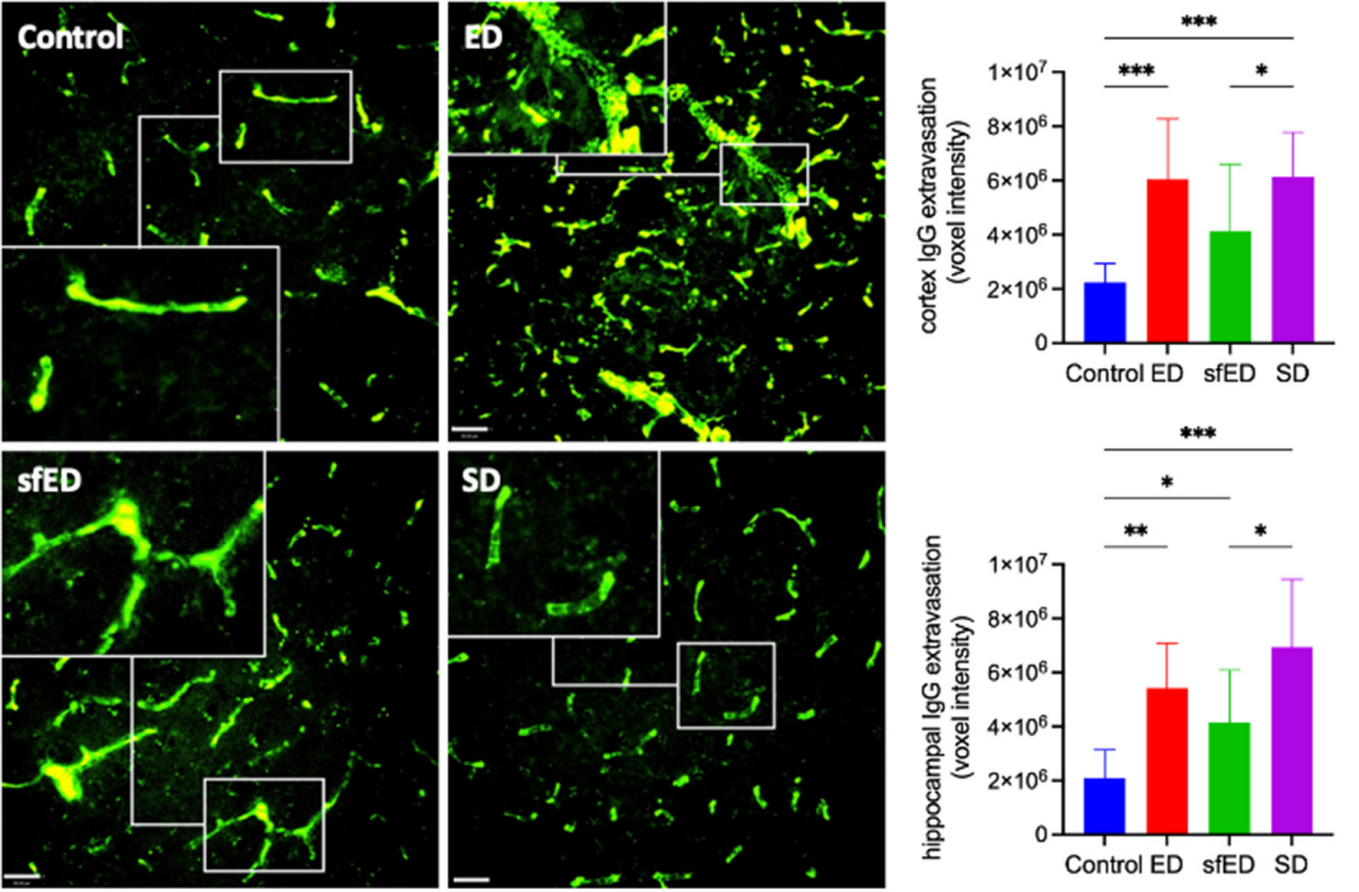
Cerebrovascular integrity measures. Twenty μm sections of brain tissue for each treatment group were stained for IgG (green) and measured under a confocal fluorescent microscope. A representative image of cortex from each group is shown for both stains at low power magnification (20x) (scale bar = 20 μm). The IgG images are accompanied by a high power magnified image (40x) in the white rectangular sub-frame within each low power image. All measures are of voxel intensity and therefore render the data to show relative levels rather than absolute concentrations. Statistical analysis was performed using Kruskal-Wallis test with uncorrected Dunn's *post-hoc* analysis (*n* = 10; *p < 0.05, **p < 0.005, ***p < 0.0005).

Cerebrovascular BBB permeability was assessed by determining brain parenchymal extravasation of IgG. Figure 1 and Supplementary Figure 2 depict IgG abundance within cortex (CTX) and hippocampal formation (HPF). Mice maintained on ED demonstrated twice the abundance of parenchymal IgG compared to control mice maintained on water alone, indicating significant breakdown of BBB. However, the amplification effect of ED treatment on parenchymal IgG was comparable to mice provided SD. Mice maintained on sfED also had exaggerated parenchymal IgG, although this effect was less than that realized in either ED or SD treated mice.

**Figure 2:**
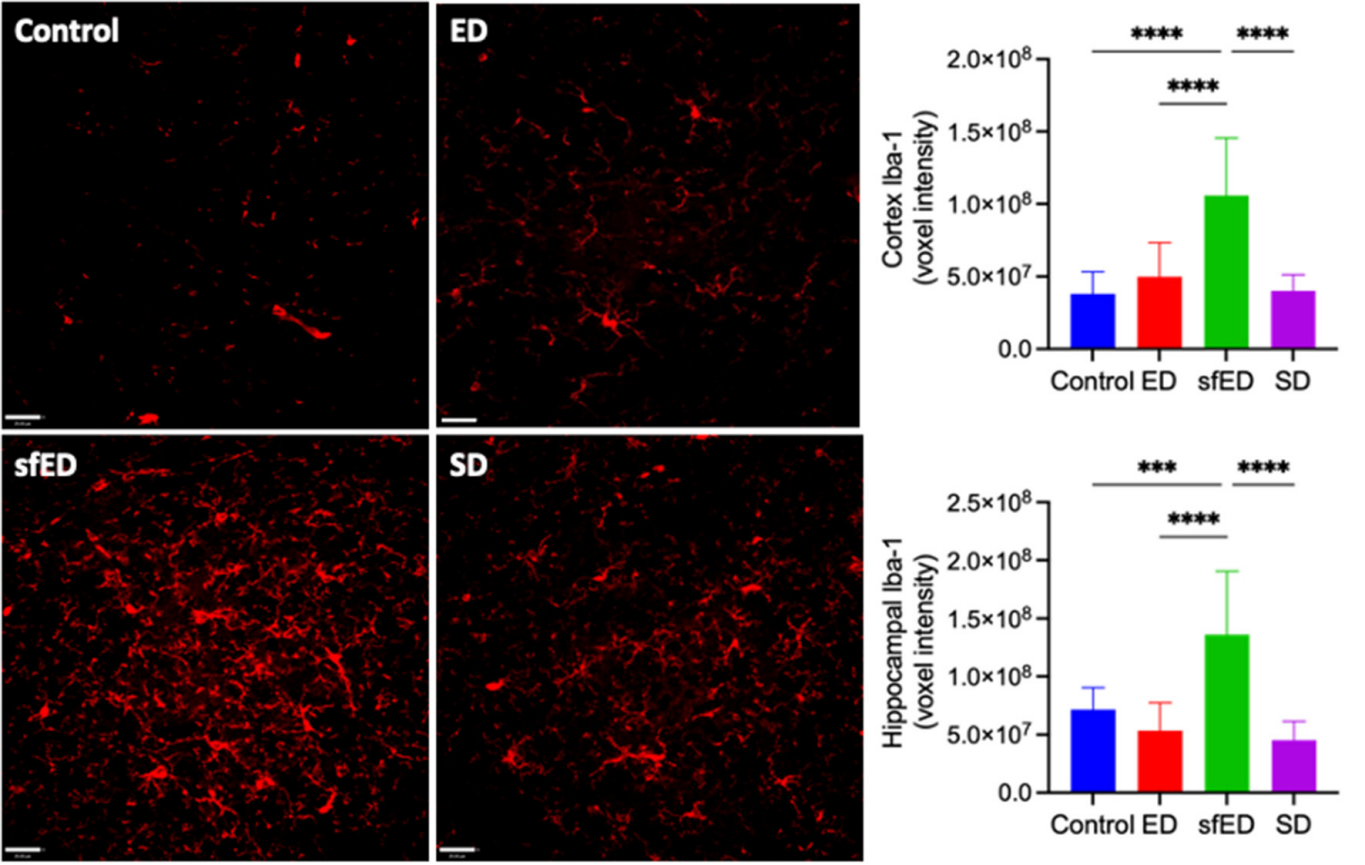
Neuroinflammatory measures. Twenty μm sections of brain tissue for each treatment group were stained for Iba-1 (red) and measured under a confocal fluorescent microscope. A representative image of each group is shown for both stains at low power magnification (20x) (scale bar = 20 μm). All measures are of voxel intensity and therefore render the data to show relative levels rather than absolute concentrations. Statistical analysis was performed using one way ANOVA with Fisher's LSD *post-hoc* analysis (*n* = 10; ***p < 0.0005; ****p < 0.0001).

Neuroinflammation was assessed by quantitatively measuring Iba-1, a sensitive marker of microglial activation. Increased BBB permeability in ED and SD treated mice did not result in heightened neuroinflammation compared to the control mice (Figure 2 and Supplementary Figure 3). However, mice maintained on the sfED had markedly exaggerated microglial activation.

**Figure 3:**
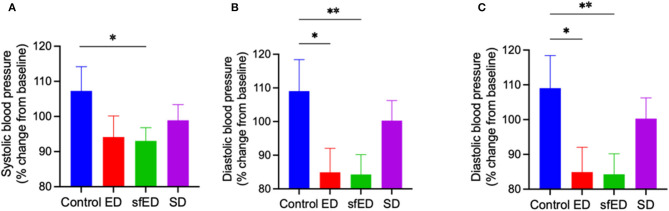
Blood pressure. The mice received Mother (ED), sugar-free Mother (sfED), Coca-Cola (SD), or water (Control) for 13 weeks. The change in systolic blood pressure **(A)**, diastolic blood pressure **(B)**, and mean arterial pressure **(C)** between baseline and at the end of the experiment (13 weeks) were measured using a tail cuff method. Statistical analysis was performed using a one-way ANOVA with Fisher's LSD *post-hoc* analysis (*n* = 10; *p < 0.05, **p < 0.005).

The effects of EDs and SD on blood pressure are provided in Figures 3A–C. The mice maintained on ED for 13 weeks showed decreasing trend in systolic blood pressure compared to the control mice receiving water (Figure 3A). The reduction of diastolic pressure and mean arterial pressure in ED mice was significant (Figures 3B,C). In sfED treated mice, there was a significant reduction of systolic pressure compared to the control mice. Similar significant reductions were indicated for diastolic pressure and mean arterial pressure compared to the control group. In SD mice, the blood pressure was comparable to the control mice.

**Figure 4:**
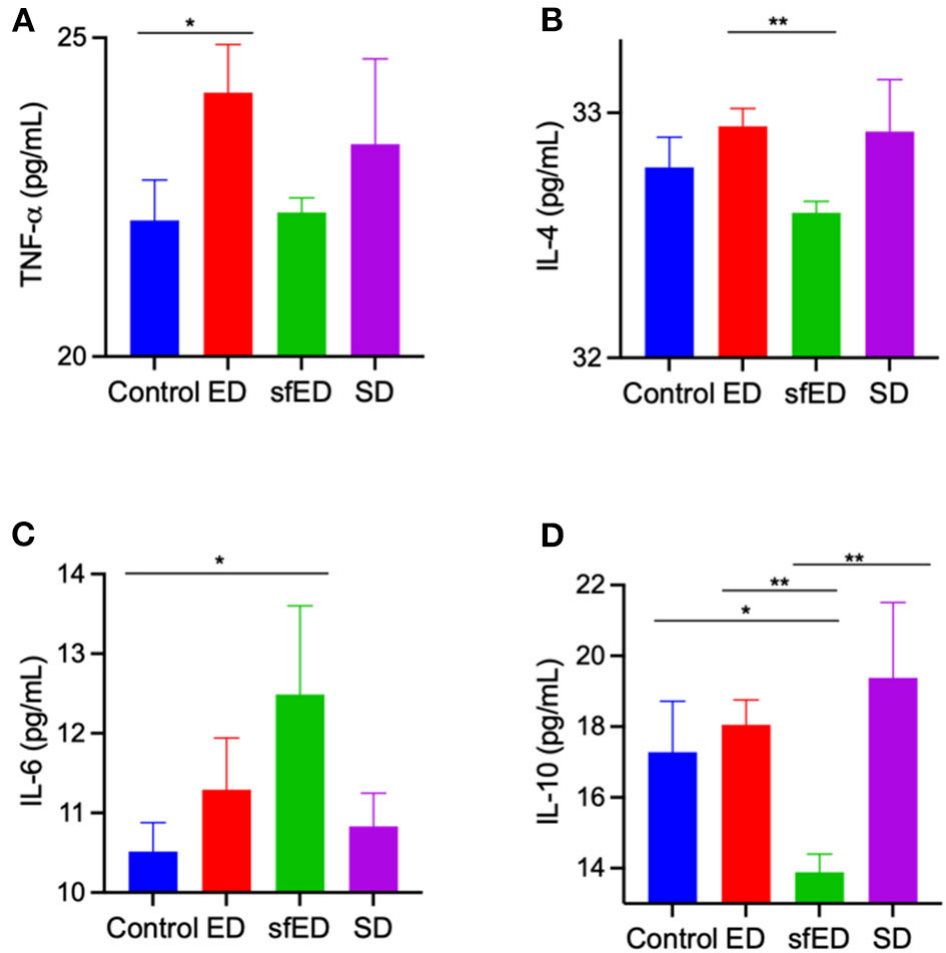
Serum cytokine concentrations. The mice received Mother (ED), sugar-free Mother (sfED), Coca-Cola (SD), or water (control) for 13 weeks. Serum concentrations of inflammatory cytokines, TNF-α **(A)**, IL-4 **(B)**, IL-6 **(C)**, and IL-10 **(D)** were measured using the flow cytometry beads array. Statistical analysis was performed Kruskal-Wallis test with uncorrected Dunn's *post-hoc* analysis (*n* = 10; *p < 0.05, **p < 0.005).

The serum concentration of inflammatory cytokines, IL-4, IL-10, and TNF-α were determine (Figures 4A–D). TNF-α, a pro-inflammatory cytokine, was modestly increased in ED treated mice compared to control mice and no other significant differences were realized for IL-4, IL-6, or IL-10. The plasma inflammatory cytokine in SD group was also unremarkable. In sfED treated mice where inflammation was realized, TNF-α was not exaggerated but rather, sfED mice had lower levels of IL-4 and IL-10 and elevated IL-6.

## Discussion

Previous studies show detrimental effects of energy drink consumption on brain function. Diaz et al. demonstrated in rats, substantial inflammation and oxidative stress within the hippocampus and temporal cortex following the intake of energy drinks (34). Furthermore, the ingestion of energy drinks resulted in neurodegeneration and astrocytosis in hippocampal CA1 and dentate gyrus regions of adult rats (29). However, the mechanisms whereby the energy drink consumption compromise cerebral function and integrity are presently unknown. In the current study, the putative effects of chronic ingestion of energy drink, Mother^TM^ (ED) and sugar free Mother^TM^ (sfED) in comparison to Coca-Cola^TM^ (SD) on neurovascular integrity and neuroinflammation were determined in wild-type C57BL/6J male mice. The higher energy intake through the ED or SD drink interventions was compensated by the lowered chow consumption, which resulted in the comparable total energy intake and weight gain across all intervention groups including the control group receiving only water. Nonetheless, the intake of ED, SD and indeed sfED led to a significant increase in blood glucose.

Following the 13 week drink interventions, this study demonstrated substantially greater BBB permeability in the mice that were maintained on ED, compared to the water-fed control mice. However, the exaggerated parenchymal abundance of IgG in ED mice was comparable to SD group mice. The latter observation suggests that the combination of taurine, B-group vitamins and significantly greater caffeine in the ED beverage does not exacerbate the BBB dysfunction induced by an ingestion of SD *per se*. Nonetheless, the experimental design adopted in this study cannot rule antagonistic components of ED differentially modulating BBB function. The sfED treated mice also had exaggerated parenchymal IgG, although less substantial than ED or SD. The data suggest that the provision of significant amounts of sucrose in ED and SD beverages contribute in part to the disruption of cerebral capillary BBB integrity.

Increased BBB permeability indicated in ED and SD treated mice did not result in neuroinflammation as indicated by the surrogate marker, Iba-1. The observation suggests that the degree of BBB dysfunction is modest and insufficient to activate astroglial cells. Rather, mice provided with sfED had marked microglial activation. One potential mechanism for the differential effects of ED vs. sfED is that the provision of the artificial sweetener including sucralose and acesulfame potassium is pro-inflammatory, or as a consequence of interactive effects with macro/micronutrients of the energy drink. The sfED formulation may also have had secondary indirect effects on neuroinflammation, via modulation of other physiological parameters. Consistent with the latter, we confirmed that chronic ingestion of ED markedly reduced blood pressures, consistent with our previous findings (17). Whilst caffeine is widely recognized to raise blood pressure, taurine has marked acute and chronic hypotensive effects (35–37). Ishikawa et al. showed that drug-induced hypotension in dogs resulted in BBB dysfunction (38). Similarly, in a rabbit model of hyper- and hypo-tension the effects on BBB integrity were found to be U-shaped, but with hypotension effects being significantly greater than the hypertension-induced BBB hyperpermeability (39).

Key specific cytokines were assessed in the context of modulation of cerebrovascular BBB integrity and neuroinflammation. Interestingly, a modest but nonetheless significant increase in TNF-α was realized in ED and to a lesser extent in SD treated mice. This may be due to AGE-RAGE interaction which occurs in hyperglycaemic conditions, consequently releasing TNF-α and inducing neuroinflmmation and oxidative stress in diabetic neuropathy (25). However, in this species with the indicated intervention with ED or SD, this was not associated with neuroinflammation *per se*. Compensatory changes in anti-inflammatory IL-4 or IL-10 were not suggested in ED or SD, with plasma concentrations comparable to control mice provided water. Rather in sfED mice where marked glial cell activation was indicated, there was modestly greater plasma abundance of the pro-inflammatory cytokine, IL-6 and reduced levels of the anti-inflammatory IL-4 and particularly IL-10. This marked reduction of anti-inflammatory cytokines may be induced by artificial sweeteners as reported previously (40). Therefore, in sfED, neuroinflammation may in part have reflected a shift to a more inflammatory plasma cytokine phenotype.

In conclusion, in a rodent model the long-term intake of ED (Mother^TM^) increased capillary permeability, but was equipotent to SD (Coca-Cola^TM^). Consumption of ED or SD had no significant effect on measures of neuroinflammation, despite some indication of modestly exaggerated pro-inflammatory cytokines. Moreover, elevation of blood pressures was not indicated, despite significant intake of caffeine. Rather, consumption of sfED induced marked microglial activation, concomitant with a substantial reduction in blood pressure and a modest change in plasma cytokines toward a more pro-inflammatory state. The differential effects of sfED and ED suggest that the artificial sweetener may be causally associated with the detrimental neuroinflammatory effects realized. However, the present study was a preliminary study and did not examine the effects of specific individual component of energy drinks on BBB. In addition, the present study did not consider sex differences. Therefore, future studies may consider these points and may attempt to identify the macro/micro-nutrient that is responsible for the disruption of BBB and increased neuroinflammation.

## Data Availability Statement

The original contributions presented in the study are included in the article/Supplementary Material, further inquiries can be directed to the corresponding author/s.

## Ethics Statement

The animal study was reviewed and approved by Curtin Animal Ethics Committee.

## Author Contributions

The study was designed by LG, VL, JM, and RT. The experiments were carried out by LG, VL, ZD'A, MN, and RT. The data collection and interpretation were done by LG, VL, ZD'A, MN, JM, and RT. The manuscript was prepared by LG, VL, MN, JM, and RT. All authors contributed to the article and approved the submitted version.

## Conflict of Interest

The authors declare that the research was conducted in the absence of any commercial or financial relationships that could be construed as a potential conflict of interest.
