# Supplementary material for: The Consumption of Energy Drinks Induces Blood-Brain Barrier Dysfunction in Wild-Type Mice
Source: Front Nutr. 2021 May 3;8:668514. doi: 10.3389/fnut.2021.668514 (PMC8126614; doi:10.3389/fnut.2021.668514)
Supplement: Supplementary file 1 [file Data_Sheet_1.docx]

Supplementary Material

Supplementary Table 1. Nutrition composition table of energy drinks, soft drink, and chow

| Per 100g or mL | **Mother** | **Sugar-free Mother** | **Coca Cola** | **AIN93M** |
| --- | --- | --- | --- | --- |
| **Energy (kJ)** | 191 | 19 | 180 | 1570 |
| **Carbohydrate, Total (g)** | 10.1 | 0.1 | 10.6 | 64.8 |
| **Fat, Total (g)** | 0 | 0 | 0 | 4 |
| **Caffeine (mg)** | 31.9 | 31.8 | 9.70 | 0 |
| **Taurine (mg)** | 400 | 400 | 0 | 0 |
| **Vitamin B3 (mg)** | 1.8 | 1.8 | 0 | 3 |
| **Vitamin B6 (mg)** | 0.2 | 0.2 | 0 | 0.7 |
| **Vitamin B12 (ug)** | 0.5 | 0 | 0 | 10.3 |
| **Vitamin B5 (mg)** | 0.66 | 0.66 | 0 | 1.65 |


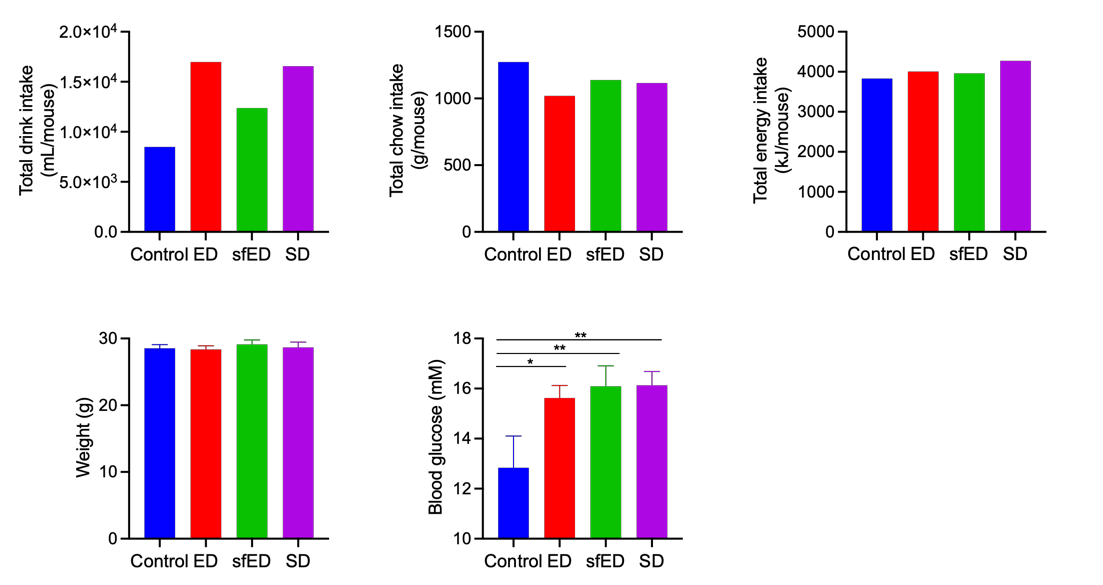


**Supplementary Figure 1.** **Weights and drink/chow intake**
The mice received Mother (ED), sugar-free Mother (sfED), Coca-Cola (SD), or water (Control) for 13 weeks. Drink and chow intake was recorded as per group as the mice were group caged. Estimated total drink intake (A), chow intake (B) and energy intake (C) per mouse are presented. Their final weight (D) and blood glucose (E) were recorded at the end of the study. Statistical analysis was performed using a one-way ANOVA with Fisher’s LSD post hoc analysis (n=10).


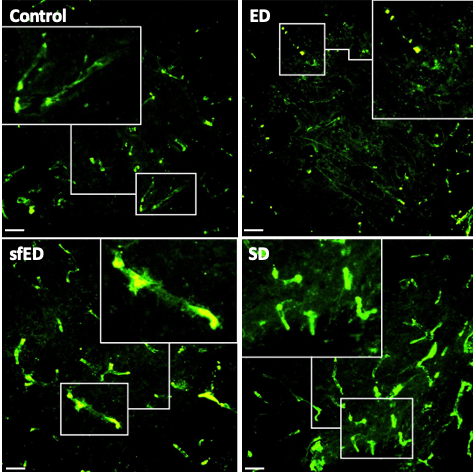


**Supplementary Figure 2.** **Cerebrovascular IgG extravasation micrographs of hippocampus**
The mice received Mother (ED), sugar-free Mother (sfED), Coca-Cola (SD), or water (Control) for 13 weeks. 20 µm sections of brain tissue for each treatment group were stained for IgG (green) and measured under a confocal fluorescent microscope. A representative image of cortex from each group is shown for both stains at low power magnification (20x) (scale bar = 20 µm). The IgG images are accompanied by a high power magnified image (40x) in the white rectangular sub-frame within each low power image.

**
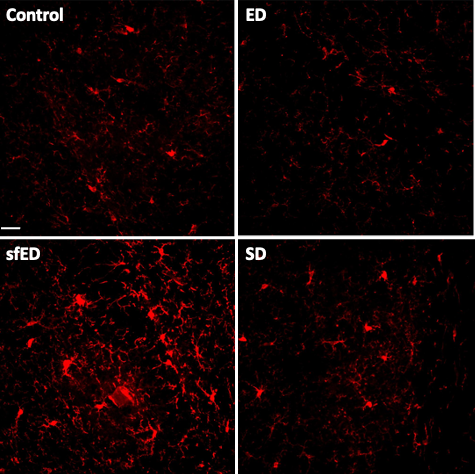
**

**Supplementary Figure 3.** **Neuroinflammatory Iba-1 micrographs of hippocampus**
The mice received Mother (ED), sugar-free Mother (sfED), Coca-Cola (SD), or water (Control) for 13 weeks. 20 µm sections of brain tissue for each treatment group were stained for Iba-1 (red) and measured under a confocal fluorescent microscope. A representative image of each group is shown for both stains at low power magnification (20x) (scale bar = 20 µm).
